# From biomarkers to mechanisms: deepening the clinical translation of inflammatory indices in colorectal cancer pathogenesis

**DOI:** 10.1097/JS9.0000000000003405

**Published:** 2025-09-11

**Authors:** Liang Yan, Biqing Yi, Yi Fang

**Affiliations:** Department of Nosocomial Infection, Guangzhou Chest Hospital Affiliated to Guangdong Pharmaceutical University, P.R. China

*Dear Editor,*

The comprehensive cross-sectional analysis conducted by Zhou *et al*, which evaluated 12 inflammatory indices in colorectal cancer (CRC) utilizing NHANES data from 2001 to 2020, offers significant epidemiological validation for the Neutrophil-to-Lymphocyte Ratio (NLR) and Lymphocyte-to-Monocyte Ratio (LMR) as non-invasive biomarkers for CRC risk. Additionally, the study establishes a correlation between the Systemic Immune-Inflammation Index (SII) and Neutrophil-to-Platelet Ratio (NPR) with mortality outcomes^[1]^. The study’s methodological rigor, particularly in adjusting for confounders and exploring nonlinear relationships, enhances the robustness of its findings. Nevertheless, the inherent limitations of the cross-sectional design restrict causal inference, and the associations observed necessitate further mechanistic investigation to facilitate the clinical application of these indices. We propose three critical extensions to optimize their translational potential.

Firstly, to advance our understanding of CRC pathogenesis, it is imperative to elucidate the biological mechanisms underlying these indices. The association between elevated NLR and increased CRC risk likely indicates a role for neutrophil-mediated tumor promotion, potentially through the formation of neutrophil extracellular traps (NETs). These structures may reactivate dormant cancer cells and facilitate metastasis via integrin activation^[2]^. A decreased LMR suggests impaired lymphocyte surveillance and monocyte-induced immunosuppression, as tumor-associated macrophages (TAMs) release cytokines such as IL-10 and TGF-β, which inhibit CD8+ T cells and recruit regulatory T cells (Tregs)^[3]^. Similarly, the association of SII and NPR with increased mortality may involve the formation of platelet-neutrophil complexes that enhance circulating tumor cell (CTC) survival through P-selectin/ICAM-1 interactions^[4]^. To substantiate these mechanisms, it is crucial to correlate NLR with circulating NET biomarkers, such as citrullinated histone H3 and cell-free DNA, and to associate LMR with TAM density in tumor biopsies. Such validation efforts would transform statistical associations into biologically substantiated tools.

Secondly, the clinical translation of research necessitates the implementation of dynamic monitoring frameworks and AI-enhanced risk stratification. Static measurements fail to account for the inflammatory changes occurring during carcinogenesis^[5]^. The integration of serial trends in NLR and LMR with fecal immunochemical tests (FIT) holds potential for improving early detection. Machine learning models that analyze temporal patterns in high-risk populations, such as those with Lynch syndrome, may surpass the accuracy of single-threshold approaches^[6]^. In terms of prognosis, the combination of SII and NPR with circulating tumor DNA (ctDNA) could enhance post-resection surveillance. Patients exhibiting high SII and detectable ctDNA following surgery, indicative of residual inflammation-driven micrometastases, might benefit from intensified adjuvant therapy^[7]^. AI algorithms that synthesize these multimodal data could generate personalized risk scores, advancing beyond traditional quartile-based stratification.

Thirdly, these indices should guide the development of targeted prevention and interception strategies. If a causal relationship is established, NLR/LMR could inform chemopreventive approaches: individuals with high NLR might benefit from CXCR2 inhibitors, such as SX-682, to inhibit neutrophil recruitment, or from low-dose aspirin to disrupt platelet-neutrophil interactions, particularly in cases of PIK3CA-mutant CRC, where aspirin has been shown to reduce incidence by 50%^[8]^. Conversely, patients with low LMR could be administered immunonutrition, including omega-3 fatty acids and arginine, to enhance lymphocyte function. Prospective clinical trials should investigate whether modulating these indices can decrease CRC incidence in high-risk populations, capitalizing on their cost-effectiveness for population screening.

In conclusion, the research conducted by Zhou *et al* establishes a solid epidemiological basis. Future investigations could aim to: (1) elucidate molecular mechanisms via paired tissue-blood analyses, (2) create AI-driven dynamic monitoring platforms, and (3) validate these indices as interception biomarkers in prevention trials. This mechanistic and translational approach has the potential to fully realize their utility in the precision management of CRC.

## Data Availability

Not applicable.
